# Evaluation of the effect of *Lepidium meyenii* Walpers in infertile patients: A randomized, double‐blind, placebo‐controlled trial

**DOI:** 10.1002/ptr.7287

**Published:** 2021-09-28

**Authors:** Ingrid Melnikovova, Daniela Russo, Tomas Fait, Michaela Kolarova, Jan Tauchen, Nataliya Kushniruk, Roberto Falabella, Luigi Milella, Eloy Fernández Cusimamani

**Affiliations:** ^1^ Department of Crop Sciences and Agroforestry, Faculty of Tropical AgriSciences Czech University of Life Sciences Prague Prague 6 Czech Republic; ^2^ Department of Science University of Basilicata Potenza Italy; ^3^ Spinoff BioActiPlants.r.l., Department of Science University of Basilicata Potenza Italy; ^4^ Department of Obstetrics and Gynecology, Second Faculty of Medicine Charles University Prague 5 Czech Republic; ^5^ Department of Agroecology and Biometeorology, Faculty of Agrobiology, Food and Natural Resources Czech University of Life Sciences Prague Prague 6 Czech Republic; ^6^ Department of Food Science, Faculty of Agrobiology, Food and Natural Resources Czech University of Life Sciences Prague Praha Czech Republic; ^7^ First Medical Faculty of Charles University Prague Prague 2 Czech Republic; ^8^ Urology Unit San Carlo Hospital, Via Potito Petrone Potenza Italy

**Keywords:** clinical trial, hormone levels, infertility, maca, semen parameters

## Abstract

Male infertility refers to the inability to conceive a natural pregnancy in a fertile female, and approximately 15% of reproductive‐aged couples worldwide face this problem. Several plants were used to treat fertility disorders and, among them, *Lepidium meyenii*, a folk medicament of Andean regions, is still used to enhance vitality and treat sterility in humans and domestic animals. The aim of the study was to evaluate the effects of *L. meyenii* Walpers on infertile patients by a randomized, double‐blind, placebo‐controlled trial. Fifty patients suffering from various reproductive‐related problems were enrolled for 16 weeks to evaluate the effect of yellow maca on semen quality and serum hormone levels. Treatment with maca improved the percentage of sperm concentration by 40%, whereas the placebo improved by 76% after 8 and 16 weeks of treatment, but the results were statistically non‐significant. No statistically significant change in hormone levels was reported by using maca, except a decrease in the level of free testosterone. Results are not sufficient to assess the efficacy of maca on male fertility. Further investigation and trials are required to obtain conclusive results.

## 1 INTRODUCTION

Infertility is described as the inability to conceive a natural pregnancy within 12 months in a couple (Dissanayake, Keerthirathna, & Peiris, 2019), and it was estimated at 15% of reproductive‐aged couples worldwide (Esteves, Hamada, Kondray, Pitchika, & Agarwal, 2012; Hwang Kathleen & Lipshultz Larry, 2011). Half of the overall infertility rates are due to males: male infertility is mainly due to factors such as impaired spermatogenesis and suboptimal semen parameters (Thomas et al., 2002).

In most underdeveloped countries, due to limited access to contemporary therapies, infertile couples use medicinal herbs. Nowadays, this natural approach is also used in developed countries due to the efficacy and safety of the herbs along with contemporary treatments (Abarikwu, Onuah, & Singh, 2020; Gerbino et al., 2016). Several plants or natural compounds are reported as showing promising properties for the management of male infertility, thus validating traditional beliefs (Abarikwu et al., 2020; Alizadeh et al., 2018; Kamal, Gupta, & Lohiya, 2003).

In this context, *Lepidium meyenii* (Walpers, 1843; cf Chacon, 1990), commonly known as maca, has been cultivated traditionally as a food crop and as a folk medicament to enhance vitality and treat sterility in humans and domestic animals (Canales et al., 1992). It is a species indigenous to the Central Andes of Peru, and nowadays it has been adapted to other countries, such as Yunnan Province in China, for large‐scale cultivation, due to the increasing demand (Chen, Li, & Fan, 2017; Melnikovova, Havlik, Fernandez, & Luigi, 2012; Tang et al., 2017; Wang & Zhu, 2019). The stimulant effect and the fertility‐enhancing properties of maca were further recognized (Ruiz, 2007). It reported libido and fertility enhancement, reduction of menopausal symptoms and erectile dysfunction and it was used to treat benign prostatic hyperplasia (Beharry & Heinrich, 2018). There are three phenotypes of edible maca: yellow, red, black; black maca is the rarest variety, whereas yellow maca is the most common and less expensive. The variety could influence the physiological effects of maca, due to the additional bioactive compounds.

Quality standardization of the maca product is based on the content of unique secondary metabolites called macamides, biologically active substances, which are associated, among other tonic effects, with the enhancement of male fertility (McCollom, Villinski, McPhail, Craker, & Gafner, 2005; Muhammad, Zhao, Dunbar, & Khan, 2002; Zhao, Muhammad, Dunbar, Mustafa, & Khan, 2005).

Up to now, many studies and reviews on the medicinal effects, including fertility properties, of maca have been published (Beharry & Heinrich, 2018; Peres et al., 2020; Tafuri et al., 2019). In rats and mice, maca increased sperm count, sperm motility, male sexual behaviour and prevented testosterone‐induced prostatic hyperplasia (Cicero et al., 2002; Cicero, Bandieri, & Arletti, 2001; Gonzales et al., 2005; Gonzales et al., 2006; Gonzales, Gasco, Malheiros‐Pereira, & Gonzales‐Castañeda, 2008; Sanchez‐Salazar & Gonzales, 2018; Zhang, Zhou, & Ge, 2019). In mice, maca increased embryo survival and the number of offspring (Ruiz‐Luna et al., 2005), as it did in guinea pigs (Alvarez, 1993). In fish, maca increased embryo survival, and in bulls and stallions, it improved sperm quantity and quality (Del Prete et al., 2018; Lee et al., 2004).

Even though there are a number of reports showing the effects of maca on semen quality parameters in animals, only three randomized clinical trials showed the effect of maca on men (Lee, Lee, You, & Ha, 2016). One of them showed favourable effects on sperm mobility in infertile patients (Poveda et al., 2013) and two showed positive effects of maca on several semen quality parameters in men with standard fertility (Kim, 2011; Melnikovova, Fait, Kolarova, Fernandez, & Milella, 2015). Moreover, two uncontrolled observational studies also suggested favourable effects of maca on semen quality (Gonzales et al., 2001; Tancara et al., 2010). Nowadays, there are few clinical studies assessing the efficacy of maca in improving semen quality in both infertile and fertile men. In addition to a previous study by our team (Melnikovova et al., 2015), our research aimed to prove or disprove the claim that male infertility has become a growing social problem.

## 2 MATERIAL AND METHODS

### 2.1 Subjects

The study was conducted between March 2015 and June 2016. The standard formula suggested for clinical trials was used by considering a study power of 80%, and Type 1 error of 5% (α = 0.05) and Type 2 error of 20% (β = 0.20) to calculate sample size. Sample size was calculated based on the sperm concentration, calculating 22 patients per each group (control group and the trial group supplemented by maca). Considering 10% dropouts, the final sample size was 25 participants in each group. All 50 infertile patients met the inclusion criteria and were enrolled in this single‐centre, randomized, double‐blind, placebo‐controlled, parallel trial to determine the effect of maca on semen quality and serum hormone levels. Inclusion criteria were infertile men aged between 28 and 52 years who had for 1 year unprotected intercourse failing to get pregnant and impaired semen quality. Semen characteristics as a sperm count of less than 20 million per millilitres or morphologically normal sperm <30% or reduced semen volume were considered eligible for the study. For 3 months before and during the study, all the included patients avoided any hormonal treatment, anabolic or other medical substances, to prevent serum hormone level changes. Patients with additional causes of infertility, alcohol or drug dependent were not accepted to the study. In selected participants failure of fertility was caused by oligoteratozoospermia (17 men), followed by oligoasthenoteratozoospermia (8 men) with oligozoospermia (8 men), teratospermia (4 men), asthenoteratozoospermia (3 men) and finally by asthenozoospermia, asthenospermia, azoospermia and oligoasthenozoospermia (one man per each). Six men were not able to give samples of semen for personal and health reasons at the very beginning of the study.

All patients were informed about the possible benefits and risks of the study, and they signed an agreement to join the clinical trial; the approval was obtained from an independent Ethics Committee of the General University Hospital in Prague (1158/14S‐IV). All 50 patients were enrolled from the Assisted Reproduction Centre of the Gynecological‐Obstetric Department, First Faculty of Medicine of Charles University in Prague. The involvement of the Assisted Reproduction Centre ensured higher cooperation rates and avoided selection bias.

### 2.2 Maca and placebo preparation

As a supplement, we used gelatinized and powdered dried bulb of yellow maca (*L. meyenii* Walp.) of less than 7% humidity, provided by Peruvian company Andean Roots Ltd in 2012. A placebo consisting of milled apple fibre (Country Life) and sucrose (Cukrovar Vrbatky a.s.) in the ratio 3:2 was selected for the colour and taste similarity to maca powder. The preparation of the supplement and placebo followed the same methodology as reported in our previous study (Melnikovova et al., 2015). A slight difference was the addition of a little content of silica, an anti‐caking agent, because the capsules were prepared by using a filling machine, not manually.

### 2.3 Chemical composition of maca powder

#### 2.3.1 Solvents and standards

Acetonitrile (HiPerSolv Chromanorm of LC–MS grade) was purchased from VWR Chemicals (Radnor, USA). Petroleum ether (per analysis; 40–65%) was purchased from PENTA chemicals (Prague, Czech Republic). Trifluoracetic acid (TFA) and standards of linolenic and linoleic acids were purchased from Sigma‐Aldrich (Prague, Czech Republic).

#### 2.3.2 Extraction of maca metabolites and HPLC‐UV analysis

Approx. 1 g of powdered plant material was extracted at room temperature in 10 ml petroleum ether on a laboratory shaker for 24 hr. Extracts were subsequently filtered and evaporated to dryness at 40°C under a stream of nitrogen. Residues were then re‐dissolved in petroleum ether at a concentration of 10 mg/ml, transferred to vials and submitted to HPLC‐UV analysis. For quantitative analysis, five samples of the same raw material were extracted as indicated above.

The analyses were performed by UltiMate 3000 UHPLC system (Thermo Fisher Scientific, Waltham, USA) equipped with UV/Vis detector (DAD). Separation of macamides and fatty acids was performed on an ACE Excel 5 μm SuperC18 column (250 mm× 4.6 mm, 90 Å; Aberdeen, Scotland). Gradient elution was carried out employing mobile phase A (water with 0.1% TFA) and B (acetonitrile) as follows: 0 min, 20:80 (A:B); 24 min, 0:100; 30 min, 0:100; 31 min, 20:80; and 32 min, 20:80. Injection volume was set at 20 μl, flow rate at 0.8 ml/min and column temperature at 40°C (Braca et al., 2018).

UV absorption was monitored at wavelengths between 190 and 400 nm and quantification was done at 210 nm. Evaluation of acquired data was performed in Chromeleon Software 7.2 (Thermo Fisher Scientific). Standard calibration curves of linoleic and linolenic acids were obtained in a concentration range of 100–2 μg/ml using six concentrations levels (100, 50, 20, 10, 5 and 2 μg/ml). UV peak areas of the standards were plotted against the corresponding standard concentrations using weighed linear regression to generate standard curve. Since standards of macamides are largely unavailable, they were identified by comparison of retention times and UV spectra according to previously published studies performed on macamides (McCollom et al., 2005; Melnikovova et al., 2012). Macamides were quantified by plotting their peak areas against calibration curve obtained for linoleic acid. Calibration curves of both linoleic acid and linolenic acid were very similar (curve equation for linoleic acid: *y* = 0.6081*x* + 0.5587; *R* ^2^ = 0.9996, and for linolenic acid *y* = 0.7427*x* – 0.3312; *R* ^2^ = 0.9995). Amounts of compounds were expressed as mg/g of dried weight (DW). Total macamides were expressed as sum of the macamides in % of DW excluding the amounts of linoleic and linolenic acids.

### 2.4 Experimental design

Patients were randomly divided into two groups; first, the trial group supplemented with maca (seven capsules containing each of them 400 mg per day, equal to 2.8 g/day) and second, the control group received placebo for 16 weeks. The sequence of assignments was unknown. Assignments were placed in separated and sealed envelopes reporting the order number on the outer side, and then they were put in order. A few months before starting the recruitment of subjects, the principal investigator created this random selection.

All patients and the investigators were blinded to maca treatment or placebo groups and remained blinded until after data analysis. In order to maintain and guarantee the blindness, maca and placebo were prepared similarly in appearance. The sequence of assignments was unknown also for the personnel of the Assisted Reproduction Centre.

During the treatment period, participants were contacted by phone after the first week to check compliance and any appearance of side effects because of consuming maca.

There were no co‐interventions during the trial. There were three consequent measurements of the spermiogram during the study period: before starting the study (0 weeks), after 8 weeks of treatment and finally at the end of the study (16 weeks). Blood samples were collected before and after the trial to evaluate the effect of maca on serum hormone levels.

### 2.5 Measurement of semen parameters

The collection of semen samples was carried out by masturbation after at least 3–5 days of sexual abstinence. They were analysed at the Assisted Reproduction Centre of the Gynecological‐Obstetric Department, first Faculty of Medicine, Charles University in Prague. Parameters such as the volume of ejaculate, total sperm count, sperm concentration and normal sperm morphology were measured in accordance with World Health Organization guidelines (WHO, 2010).

### 2.6 Hormone evaluation assay

Blood samples were collected at the beginning and at the end of the treatment (16 weeks) to evaluate the effect of maca on serum hormone levels. Five reproductive hormones (luteinizing hormone, follicle‐stimulating hormone, prolactin, estradiol and testosterone) and two thyroid hormones (free thyroxin and thyroid‐stimulating hormone) were evaluated in the Central Laboratory of the First Faculty of Medicine, Charles University in Prague by routine immune‐analytical methods.

### 2.7 Statistical analysis

Results were analysed using Statistica 13.2 software. An ITT analysis was performed. Due to the lack of homogeneity of variance assumptions in all cases (Bartlett's test), the analysis of inter‐group differences was carried out by a non‐parametric Mann–Whitney U test, whereas the data of more than two groups were analysed by a Kruskal–Wallis H test. Based on the Chi‐square distribution, a *p* value <.05 was considered statistically significant.

## 3 RESULTS

### 3.1 Chemical composition of maca

**FIGURE 1:**
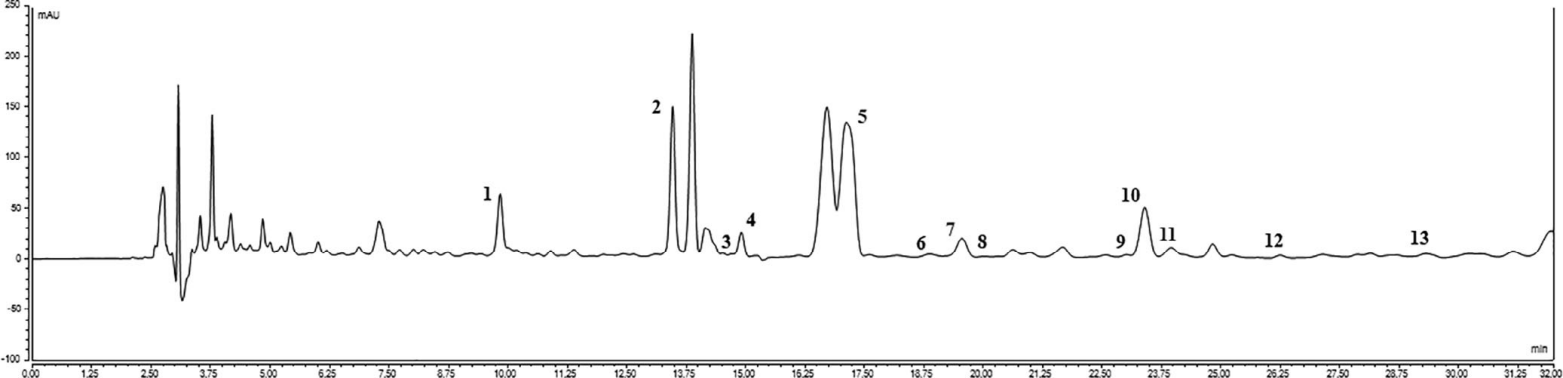
Chromatogram of maca sample: (**1**) unknown macamide; (**2**) linolenic acid; (**4**) n‐benzyl‐(9Z, 12Z, 15Z)‐octadecatrienamide; (**5**) linoleic acid; (**7**) n‐benzyl‐(9Z, 12Z)‐octadecadienamide; (**8**) n‐benzylpentadecanamide; (**9**) n‐(3‐methoxybenzyl)‐hexadecanamide; (**10**) n‐benzylhexadecanamide; (**11**) n‐benzyl‐(9Z)‐octadecenamide; (**12**) n‐benzylheptadecanamide; (**13**) n‐benzyloctadecanamide. (**3**) and (**6**) are probably methoxy‐derivatives of **4** and **7**, respectively

Maca contains unique secondary metabolites macamides and macaenes, bioactive marker compounds, which are potentially related to changes in the reproductive tract in animals and humans via modulation in semen quality. Chemical composition was carried out by HPLC‐UV analysis; Figure 1 shows the chromatogram of maca. The most abundant compounds in the petroleum ether extract of the powdered maca samples were fatty acids linoleic and linolenic acids (**2** and **5**; 72.89 and 165.65 μg/g DW, respectively). Samples contained 0.0121% (± 0.122 × 10^+^) of total macamides. The major macamides were n‐benzylhexadecanamide (**10**; 49.43 μg/g DW), unknown macamide (**1**; 32.96 μg/g DW) and n‐benzyl‐(9Z, 12Z)‐octadecadienamide (7; 18.85 μg/g DW) with smaller amounts of other structurally related macamides (**3**, **4**, **6**, **8**, **9**, **11**–**13**, Table 1) that were present at relatively lower levels, ranging from 11.56 μg/g DW to traces.

**TABLE 1:** Content of fatty acids and macamides in powdered maca samples

Peak no.	Compound^a^	Retention time (min)	μg/g DW^b^	SD^c^
1	Unknown macamide	9.78	32.96	0.77
2	Linolenic acid	13.42	72.89	11.85
3	Methoxy‐derivative of compound no. 4	14.54	<LOQ	—
4	*n*‐benzyl‐(9*Z*, 12*Z*, 15*Z*)‐octadecatrienamide	14.87	11.56	2.59
5	Linoleic acid	17.05	165.65	38.93
6	Methoxy‐derivative of compound no. 7	18.83	0.34	0.23
7	*n*‐benzyl‐(9*Z*, 12*Z*)‐octadecadienamide	19.51	18.85	3.05
8	*n*‐benzylpentadecanamide	20.63	<LOQ	—
9	*n*‐(3‐methoxybenzyl)‐hexadecanamide	22.74	<LOQ	—
10	*n*‐benzylhexadecanamide	23.50	49.43	7.29
11	*n*‐benzyl‐(9*Z*)‐octadecenamide	24.50	6.40	0.84
12	*n*‐benzylheptadecanamide	27.24	<LOQ	—
13	*n*‐benzyloctadecanamide	29.35	2.38	1.22

^a^ For details of compound identification, see Materials and Methods section.

^b^ Dry weight; data expressed as mean values (*n* = 5).

^c^ Standard deviation.

### 3.2 Patient enrolment

**FIGURE 2:**
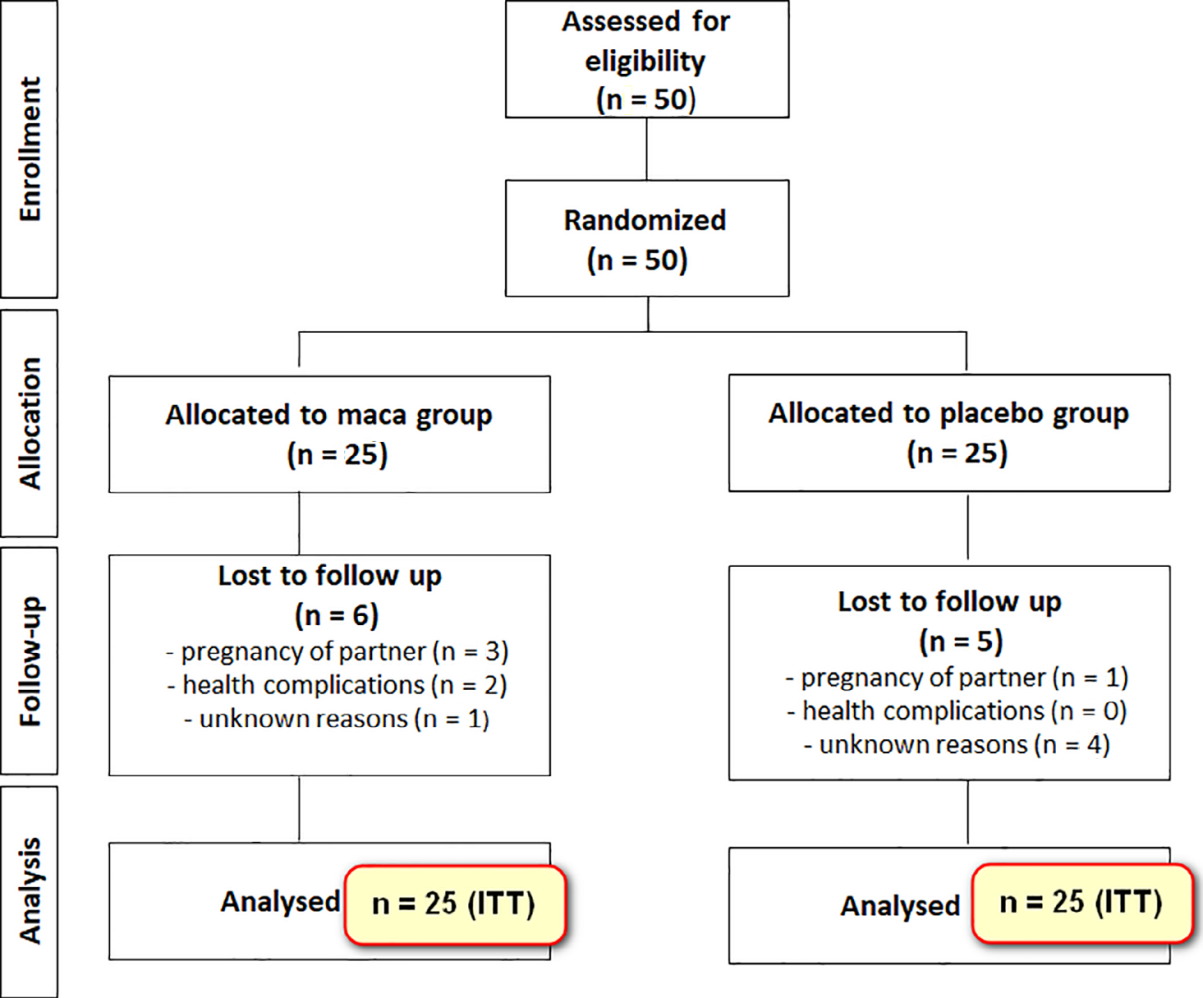
Flow diagram of enrolment, allocation, follow‐up and analysis in both groups of the study

Fifty infertile patients suffering from various combinations of oligospermia, asthenospermia, teratospermia and azoospermia were enrolled in this single‐centre, randomized, double‐blind, placebo‐controlled, parallel trial. Patients were randomly assigned to the trial and the control group (25 patients each). Eleven patients dropped out from the study, leaving 19 patients within the trial group and 20 for the placebo group. Four patients interrupted the trial because they achieved their goal – pregnancy of their partners during the follow‐up time. Three of them were from the trial group and one from the control group. Five patients dropped out for unknown reasons and two for possible adverse events of the treatment, as starch intolerance and diagnosis of ulcerative colitis. The flow diagram of the clinical trial is presented in Figure 2.

### 3.3 Semen parameters

Baseline characteristics in both groups were comparable; selected patients reported no significant differences in age, physical conditions or clinical stage of disability.

**FIGURE 3:**
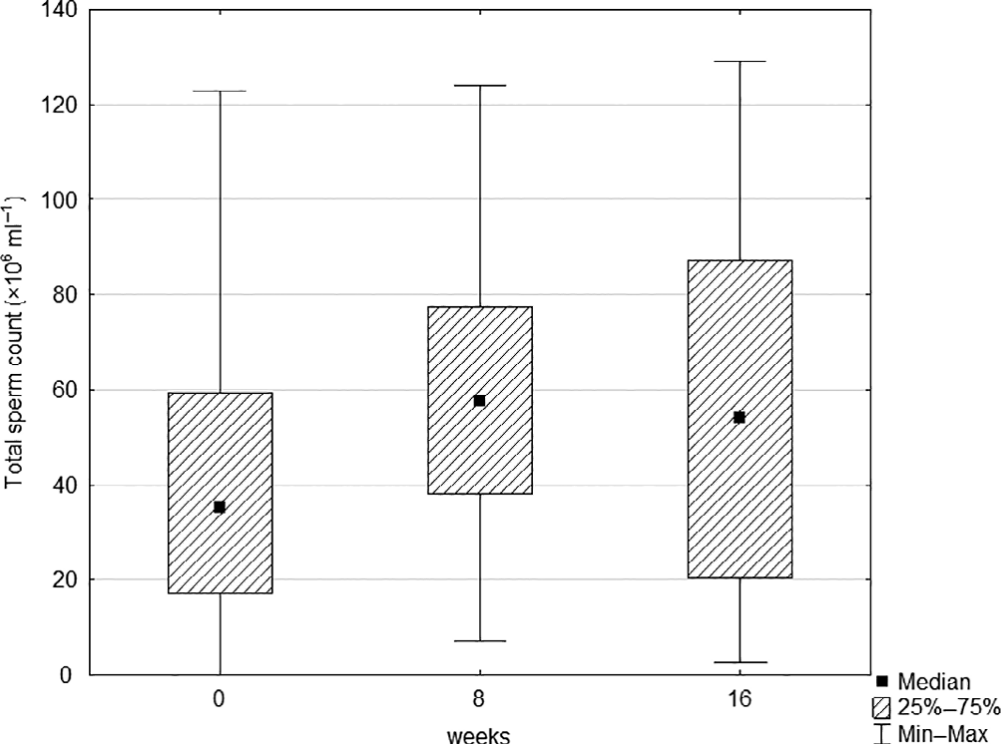
Box and whisker plot of the effect of maca treatment on total sperm count (the median, the 25th and 75th percentiles, and the minimum and maximum observed values that are not statistically outlying)

**FIGURE 4:**
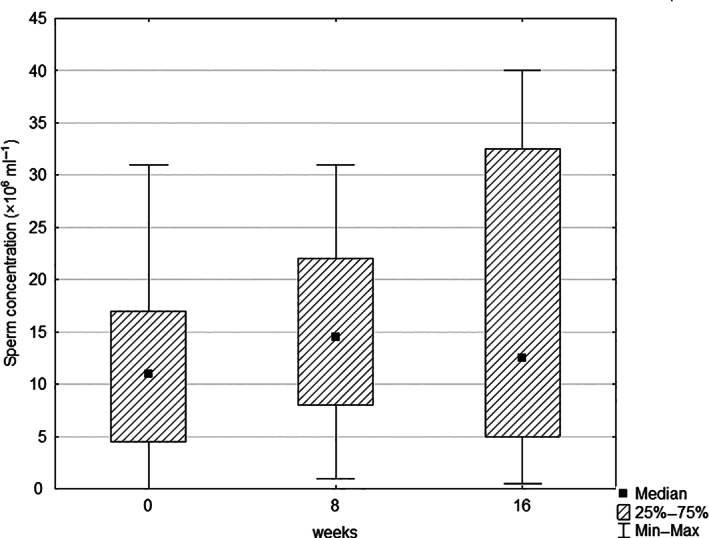
Box and whisker plot of the effect of maca treatment on sperm concentration (the median, the 25th and 75th percentiles, and the minimum and maximum observed values that are not statistically outlying)

At the beginning, the evaluation of the analysed semen parameters in the maca group reported no statistically significant differences when compared with the placebo group, probably due to the higher within‐group variation. Not significant, but a noticeable increase in various parameters was observed after 8 and 16 weeks of treatment in the maca group and in the placebo group (Tables 2 and 3). An increase during the treatment was noted at total sperm count and sperm concentration in both the maca and the placebo group, while the placebo group showed better performance, even though not statistically different. After the maca treatment, the total sperm count rose by 15% (Figure 3), and sperm concentration by 40% (Figure 4). In the placebo group, total sperm count rose by 102%, and sperm concentration by 76%.

**TABLE 2:** Mean semen parameter values during maca and placebo treatment (mean ± standard error; Kruskal–Wallis H test)

		Total sperm count (×10^6^)	Sperm concentration (×10^6^ ml^−1^)
Maca	0 weeks	54.52 ± 12.24	12.32 ± 2.14
	8 weeks	83.01 ± 22.37	17.64 ± 3.50
	16 weeks	62.68 ± 13.00	17.31 ± 3.55
	*p value*	.29	.43
	*H*	2.49	1.67
Placebo	0 weeks	36.80 ± 12.24	10.05 ± 2.14
	8 weeks	65.16 ± 22.37	16.79 ± 3.50
	16 weeks	74.49 ± 13.01	17.69 ± 3.55
	*p value*	.16	.17
	*H*	3.69	3.55

**TABLE 3:** Comparison of parameters at the beginning and the end of the study (ITT analysis)

Group		Percentage of all 25 patients in each group (ITT)	
		Total sperm count (×10^6^)	Sperm concentration (×10^6^ ml^−1^)
Maca	Increase	48%	48%
	Decrease	28%	24%
	Same	0%	4%
Placebo	Increase	52%	48%
	Decrease	28%	32%
	Same	0%	0%

A statistically significant decrease in the level of free testosterone between the baseline and 16 weeks of treatment of the maca group was found. The average level decreased by 27%. At the end of maca or placebo treatment, no substantial changes of other hormone levels were observed in the blood serum after 16 weeks of maca or placebo administration (Table 4).

**TABLE 4:** Baseline and post‐treatment serum level of hormones in maca and placebo‐treated subjects (mean ± standard error; Mann–Whitney *U* test)

		LH	FSH	Prolactin	Estradiol	Testosterone	Free testosterone
Maca	0 weeks	2.56 ± 0.32	4.42 ± 0.66	11.89 ± 3.6	0.08 ± 0.01	16.21 ± 1.77	0.11 ± 0.02
	16 weeks	3.75 ± 0.89	4.72 ± 0.93	11.92 ± 3.37	0.08 ± 0.01	17.61 ± 1.28	0.06 ± 0.02
	*p value*	.38	.63	1.00	.60	.44	.04 ± .02
Placebo	0 weeks	2.89 ± 0.26	3.18 ± 0.22	8.55 ± 1.42	0.08 ± 0.01	16.25 ± 2.93	0.15 ± 0.07
	16 weeks	3.00 ± 0.89	3.31 ± 0.33	10.87 ± 1.31	0.07 ± 0.01	14.43 ± 1.80	0.03 ± 0.01
	*p value*	.65	.96	.33	.38	.79	.07

Abbreviations: *FSH*, follicle‐stimulating hormone; *LH*, luteinizing hormone.

## 4 DISCUSSION

The target of the present double‐blind placebo‐controlled trial was to find scientific evidence of maca impact on spermatogenesis. Maca contains a group of secondary metabolites such as macamides, macaenes and other lipid fractions that are potentially related to changes in the reproductive tract via modulation in semen quality (Gasco, Aguilar, & Gonzales, 2007; Melnikovova et al., 2012; Y. Wang, Wang, McNeil, & Harvey, 2007). The content of these compounds was analysed by HPLC‐UV system, confirming the presence of linoleic and linolenic acids and 0.0121% (± 0.122 × 10^−5^) of total macamides, in accordance with previously published study (McCollom et al., 2005). The main known macamides were n‐benzylhexadecanamide (**10**; 49.43 μg/g DW) and n‐benzyl‐(9Z, 12Z)‐octadecadienamide (**7**; 18.85 μg/g DW).

To the best of our knowledge, our trial represents one of the few clinical trials using maca to improve the semen quality parameters. In the previous pilot study, Melnikovova et al. (2015) reported improvement of semen parameters after 12 weeks of 1.75 g/day maca administration. We increased the dose to the pilot study and subjects consumed 2.8 g/day of powdered maca at doses of seven tablets with 40 mg each. We also prolonged the duration of maca intake by 4 weeks to a final 16 weeks, following the literature data reporting the time and dose‐dependent effect of maca. Gonzales et al. (2001) reported an increase of sperm production and sperm motility after treatment with maca, but only nine men received tablets of maca (1.5 or 3.0 g/day) for 4 months, and a placebo was not included. Administration of powder maca (1.75 g/day) for 12 weeks in 18 adult men showed a rising trend in the maca group in sperm motility and sperm count compared with placebo (Melnikovova et al., 2015). No placebo was used in the trial carried out by Tancara et al. (2010) on 10 adults with alteration in semen parameters; the pulverized and dehydrated maca root in the capsule (3 g/day) improved sperm motility. The randomized, double‐blind, placebo‐controlled study (data published as abstract only) showed that the oral supplementation of maca extract (NatureWay Products, Inc.), 2 g/day, could improve seminal parameters such as concentration and motility of sperm of infertile patients (Lee et al., 2016).

In our double‐blind placebo‐controlled trial, we have observed some irrelevance in the data obtained compared with our review of the literature. According to Alcalde and Rabasa (2020), sperm concentration increased in infertile adult men treated with maca compared with placebo. Our results reported as the total sperm count increased by 15% in patients using maca for the period of 16 weeks, but we noticed doubling of total sperm count in the placebo group. Moreover, there was an increase in semen concentration by 40% and by 76% in the trial group and the control group, respectively. The results are contrary to our pilot study from 2015 (Melnikovova et al., 2015) and our review of the literature too. No change in hormone levels but a decrease in free testosterone was observed, and our data confirmed previous results in pre‐clinical and clinical studies (Gonzales et al., 2001; Gonzales, Rubio, Chung, Gasco, & Villegas, 2003; Melnikovova et al., 2015; Santos, Howell, & Teixeira, 2019; Tancara et al., 2010), suggesting the stimulation of sperm quality could happen by a non‐steroidogenetic mechanism.

To date, clinical evidence is scarce, but several in vivo studies to improve male fertility were carried out on experimental models. For animal experiments, different preparations were administered, whereas for clinical trials, maca powder was usually used. The preparation of aqueous extracts by boiling and filtration following traditional culture in the high Andes was widely used (Bustos‐Obregón, Yucra, & Gonzales, 2005; Chung, Rubio, Gonzales, Gasco, & Gonzales, 2005; Gonzales et al., 2001; Gonzales et al., 2004; Gonzales et al., 2005; Gonzales et al., 2006; Rubio et al., 2006), but other extracts such as hexane, chloroform, methanol, ethanol, etc. (Cicero et al., 2002; Gonzales et al., 2003; Gonzales et al., 2008; Inoue, Farfan, & Gonzales, 2016; Ohta et al., 2016; Yucra, Gasco, Rubio, Nieto, & Gonzales, 2008; Zheng et al., 2000), and maca powder were also tested (Cicero et al., 2001; Clément, Kneubühler, Urwyler, Witschi, & Kreuzer, 2010; Lavana, Vazquez, Palma‐Irizarry, & Orihuela, 2013).

Interesting evidence was obtained in experimental models; the administration of maca to animals improved sperm quality and spermatogenesis (Chung et al., 2005; Gasco et al., 2007; Carla Gonzales et al., 2006; Gonzales et al., 2004; Gonzales et al., 2003; Sanchez‐Salazar & Gonzales, 2018; Yucra et al., 2008; Zhang et al., 2019). The effects on spermatogenesis were more evident after administration in rats of black maca extracts than treatment with extracts derived from yellow and red varieties. Onaolapo, Oladipo, and Onaolapo (2018) investigated the effect of maca (500–1,000 mg/kg for 28 days) on cyclophosphamide (CYP)‐induced gonadal toxicity in male mice and it has shown the beneficial mitigation effect of maca on CYP‐induced gonadal toxicity and subfertility. Carla Gonzales et al. (2006) reported that the administration of 666.6 mg/day (1.66 g/kg BW) of aqueous extracts from black and, to a lesser extent, yellow maca increased the length of Stage VIII and epididymal sperm count (ESC) in rats after 7 and 42 days of treatment. The long‐term treatment of 42 days with black maca extract showed an increase in daily sperm production (DSP) and a significant improvement in sperm motility in rats (Gonzales et al., 2006). No effect on sperm motility was noted in rats treated with yellow maca aqueous extract, but a dose‐related effect was confirmed increasing Stage VIII in rats with 0.01 g/kg BW of yellow maca aqueous extract until it reached a plateau at 1 g/Kg BW of extract (Chung et al., 2005). Gasco et al. (2007) confirmed that ESC was increased in adult male rats with black and yellow maca extracts after treatment of 1 g of maca/Kg BW for 84 days. The use of red maca aqueous extract reported no effect on testicular and epididymal weight and also on epididymal sperm motility and sperm count (Gasco et al., 2007; Carla Gonzales et al., 2006), but its extract reduced prostate size in rats with testosterone‐induced prostatic hyperplasia, unlike black maca, which had no effect (Gonzales, Leiva‐Revilla, Rubio, Gasco, & Gonzales, 2012).

Ethyl acetate and chloroform fractions of black maca demonstrated a higher DSP and epididymis sperm count than petroleum ether, n‐butanol and hydroalcoholic fractions after 7 days at the dose of 1 g/kg BW (Yucra et al., 2008).

Inoue et al. (2016) examined methanolic and aqueous extracts of yellow and black maca, showing that they were more effective than their butanolic fractions in improving spermatogenesis in mice at a dose equivalent to 1 g raw material/Kg BW (3 days).

Aqueous maca extract at a dose of 2.2 g/Kg daily in rats was also found to reverse the damaging effect of lead acetate on Stages VII, VIII and IX–XI on seminiferous epithelium length and maintained corpus and cauda epididymis sperm count, daily sperm count and spermatid count (Rubio et al., 2006). A study by Valdivia Cuya et al. (2016) of maca on chemically and physically induced sub‐fertility in mice also gave concurring results with 666 mg/Kg BW daily of aqueous extract. Maca was also reported to have potential use in treating erectile dysfunction related to hypogonadism and microvascular damage in diabetes (Kimura et al., 2016; Zheng et al., 2000).

Feeding with a powder hydroalcoholic extract of maca for 6 weeks increased serum testosterone concentration related to the seminal vesicle stimulation in rats, probably due to the increased ability of testosterone production by Leydig cells (Ohta et al., 2016). A previous study on stallions reported that yellow maca treatment (4 g/100 Kg BW) improved the quality and quantity of sperm and preserved progressive motility and DNA integrity, allowing more artificial insemination per ejaculate (Del Prete et al., 2018).

Despite numerous and encouraging in vivo studies on experimental models, the evidence for improving fertility in men is not sufficient, and results remain inconclusive. Some of the key points are the preparation and variety of maca, the limited sample size, the appropriate effective dose and treatment duration. Black maca seems to be more effective than other varieties on semen parameters; very high doses were used to obtain positive results, such as 2.2 g/Kg (Rubio et al., 2006) and 1.66 g/kg BW (Gonzales et al., 2004) not being practical clinically.

This study did not evidence a clinical effect related to maca supplementation that might be related to its inefficacy and/or to some other reasons. In fact, a relatively high dropout rate that resulted from reasons not related to study design, medication reducing the number of participants or influence of nutritional habit on semen quality should also be taken into account as described by Eslamian et al. (2015). For these reasons, our results indicate that further investigations testing the effects of maca should be performed, maybe by increasing the dose and the administration period.

## 5 CONCLUSIONS

In conclusion, this single‐centre, randomized, double‐blind, placebo‐controlled, parallel trial provides inconclusive evidence to assess the effects of *L. meyenii* (maca) on seminal parameters in infertile patients. No significant change in hormone level was reported after maca treatment, but a decrease of testosterone in the treated group. Our results suggest that larger‐scale trials, longer administration or higher doses are required to obtain conclusive results. Moreover, it might be taken into account that other variables such as dietary assessment, lifestyle or exposure to pollution could influence the final results.

## AUTHOR CONTRIBUTIONS

Ingrid Melnikovova, Daniela Russo and Luigi Milella designed the trial and discussed the results. Tomáš Fait and Ingrid Melnikovova conducted the clinical trial and wrote the main text of the manuscript. Eloy Fernandez designed the randomization procedure. Jan Tauchen performed the chemical characterization of maca. Michaela Kolářová performed the statistical analysis. Natalya Kushniruk helped to revise the manuscript. Daniela Russo, Ingrid Melnikovova, Roberto Falabella and Luigi Milella designed the protocol and revised the manuscript. All authors reviewed the manuscript and agreed to its submission.

## CONFLICTS OF INTEREST

The authors declared there is no conflict of interest.

## Data Availability

The data used to support the findings of this study are available from the corresponding author upon request.
